# Bacterial Vaginosis: What Do We Currently Know?

**DOI:** 10.3389/fcimb.2021.672429

**Published:** 2022-01-18

**Authors:** Linda Abou Chacra, Florence Fenollar, Khoudia Diop

**Affiliations:** ^1^ Aix Marseille Univ, IRD, AP-HM, SSA, VITROME, Marseille, France; ^2^ IHU-Méditerranée Infection, Marseille, France

**Keywords:** vaginal microbiome, *Lactobacillus*, dysbiosis, bacterial vaginosis, sexually transmitted infection, bacterial vaginosis-associated bacteria

## Abstract

The vaginal microbiome is a well-defined compartment of the human microbiome. It has unique conditions, characterized by the dominance of one bacterial species, the *Lactobacilli.* This microbiota manifests itself by a low degree of diversity and by a strong dynamic of change in its composition under the influence of various exogenous and endogenous factors. The increase in diversity may paradoxically be associated with dysbiosis, such as bacterial vaginosis (BV). BV is the result of a disturbance in the vaginal ecosystem; i.e., a sudden replacement of *Lactobacilli* by anaerobic bacteria such as *Gardnerella vaginalis, Atopobium vaginae, Ureaplasma urealyticum, Mycoplasma hominis*, and others. It is the most common cause of vaginal discharge in women of childbearing age, approximately 30% of all causes. The etiology of this dysbiosis remains unknown, but its health consequences are significant, including obstetrical complications, increased risk of sexually transmitted infections and urogenital infections. Its diagnosis is based on Amsel’s clinical criteria and/or a gram stain based on the Nugent score. While both of these methods have been widely applied worldwide for approximately three decades, Nugent score are still considered the “gold standard” of BV diagnostic tools. Given the limitations of these tools, methods based on molecular biology have been developed as alternative rational strategies for the diagnosis of BV. The treatment of BV aims at restoring the balance of the vaginal flora to stop the proliferation of harmful microorganisms. Prescription of antibiotics such as metronidazole, clindamycin, etc. is recommended. Faced with the considerable uncertainty about the cause of BV, the high rate of recurrence, the unacceptable treatment options, and clinical management which is often insensitive and inconsistent, research on this topic is intensifying. Knowledge of its composition and its associated variations represents the key element in improving the therapeutic management of patients with the most suitable treatments possible.

## 1 Introduction

The vaginal microbial community is complex and dynamic, consisting of a group of bacteria typically characterized by abundant *Lactobacilli* that evolve during the life of the woman, depending on age, hormonal estrogen levels, sexual practices and the environment (Kumar et al., 2011; Bilardi et al., 2016b). The vaginal microbiota plays a crucial role in women’s health (infection, reproduction…), and that of their fetuses (Li et al., 2012).

BV is a dysbiosis of the vaginal microbiota characterized by a shift from *Lactobacilli* dominance to that of a mixture of various anaerobic bacteria (Mårdh, 1993; Hay, 2002). It is the most common vaginal disorder worldwide in women of childbearing age (Cristiano et al., 1996; Hogan et al., 2007; Trabert and Misra, 2007). BV is associated with significant adverse healthcare outcomes, including increased susceptibility to sexually transmitted infections, urogenital infections, pelvic inflammatory disease, and an increased risk of abnormal pregnancy (Marrazzo and Hillier, 2013). The etiology of BV is still unknown. Standard antibiotic therapy often fails, with an estimated relapse rate of 50% at six months follow-up (Bradshaw et al., 2006; Bretelle et al., 2015).

## 2 Normal Healthy Vaginal Flora

The vaginal ecosystem is colonized from the very first hours of the birth of a female and remains throughout her life until death (Romero et al., 2014). Women of childbearing age produce about 1 to 4 mL of vaginal fluid, containing 10^8^ to 10^9^ bacterial cells per mL (Danielsson et al., 2011).

### 2.1 Composition of Normal Vaginal Flora

**Figure 1:**
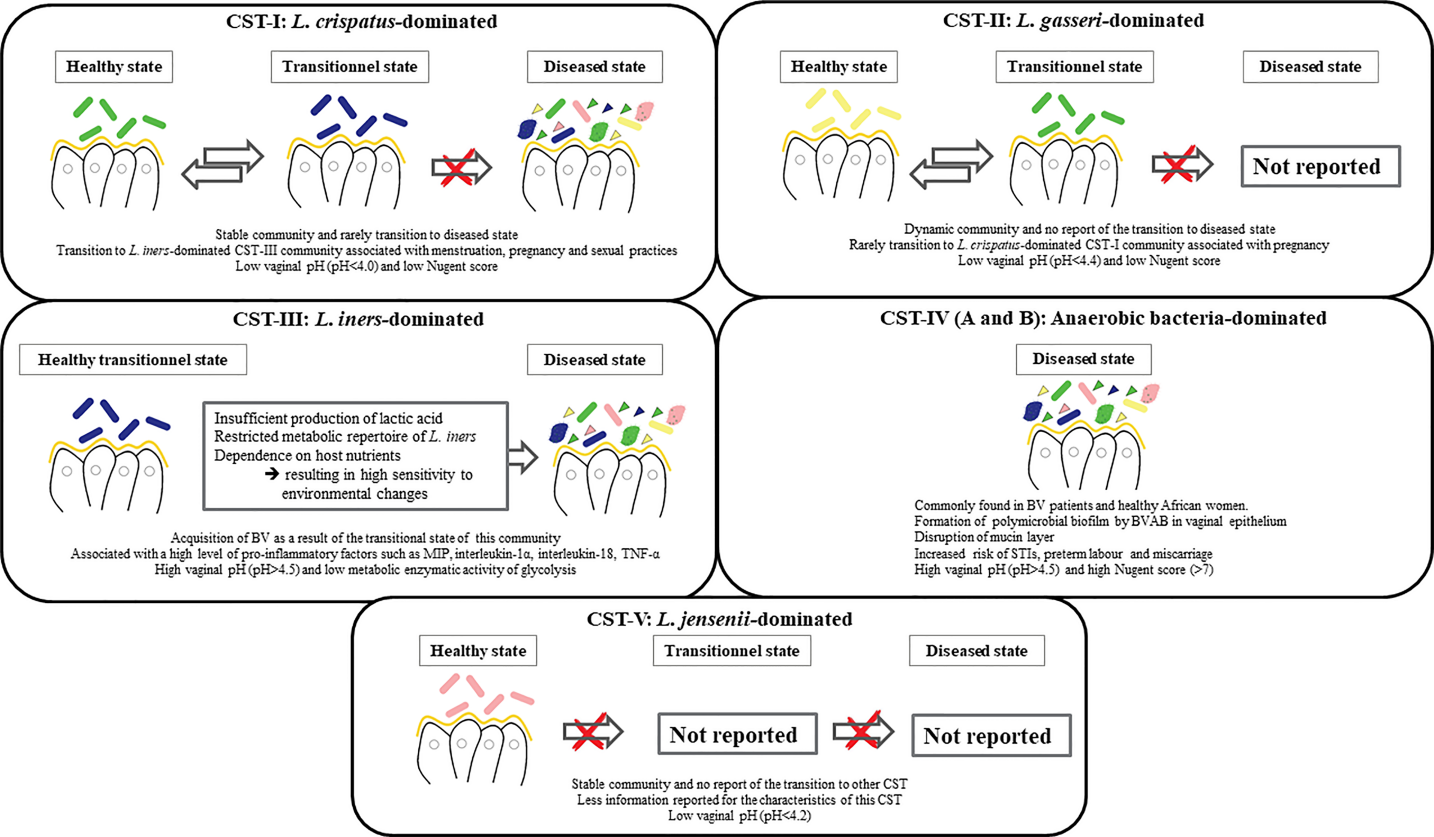
Schema illustrating the 5 vaginal community state types (CSTs).

The vaginal flora was first described by the German gynecologist Albert Döderlein in 1892, who reported a homogeneous vaginal flora of gram-positive bacilli in healthy women (Lepargneur and Rousseau, 2002). They were named “Döderlein’s bacilli” and were later identified as members of the *Lactobacillus* genus by Beijerink in 1901 (Lepargneur and Rousseau, 2002). Under normal conditions, 70-90% of the vaginal bacterial species in healthy premenopausal women are *Lactobacilli* (Africa et al., 2014). As molecular techniques have advanced, our understanding of the diversity and complexity of the vaginal bacterial community has broadened (Fredricks et al., 2005). Among more than 200 *Lactobacillus* species with standing in the nomenclature, over 20 species have been found in the vaginal flora (Huang et al., 2014). Sequencing of the 16 rRNA gene revealed that the vaginal bacterial community, mainly composed of *Lactobacilli*, is classified into five groups named community state types, namely I, II, III, IV and V (Ravel et al., 2011). Four of these groups are dominated by *Lactobacillus*. The first is dominated by *L. crispatus*, the second by *L. gasseri*, the third by *L. iners*, and the fifth by *L. jensenii*, while the fourth contains a smaller proportion of *Lactobacilli* but is composed of a polymicrobial mixture of strict and facultative anaerobes (*Gardnerella, Atopobium, Mobiluncus, Prevotella…*). Although there is always a temporal transition between vaginal bacterial communities (**Figure 1**) (Gajer et al., 2012).

Thus, many other bacteria are present at lower concentrations in healthy vaginal flora, such as *Peptostreptococcus, Bacteroides, Corynebacterium, Streptococcus*, and *Peptococcus* (Kumar et al., 2011).

**Figure 2:**
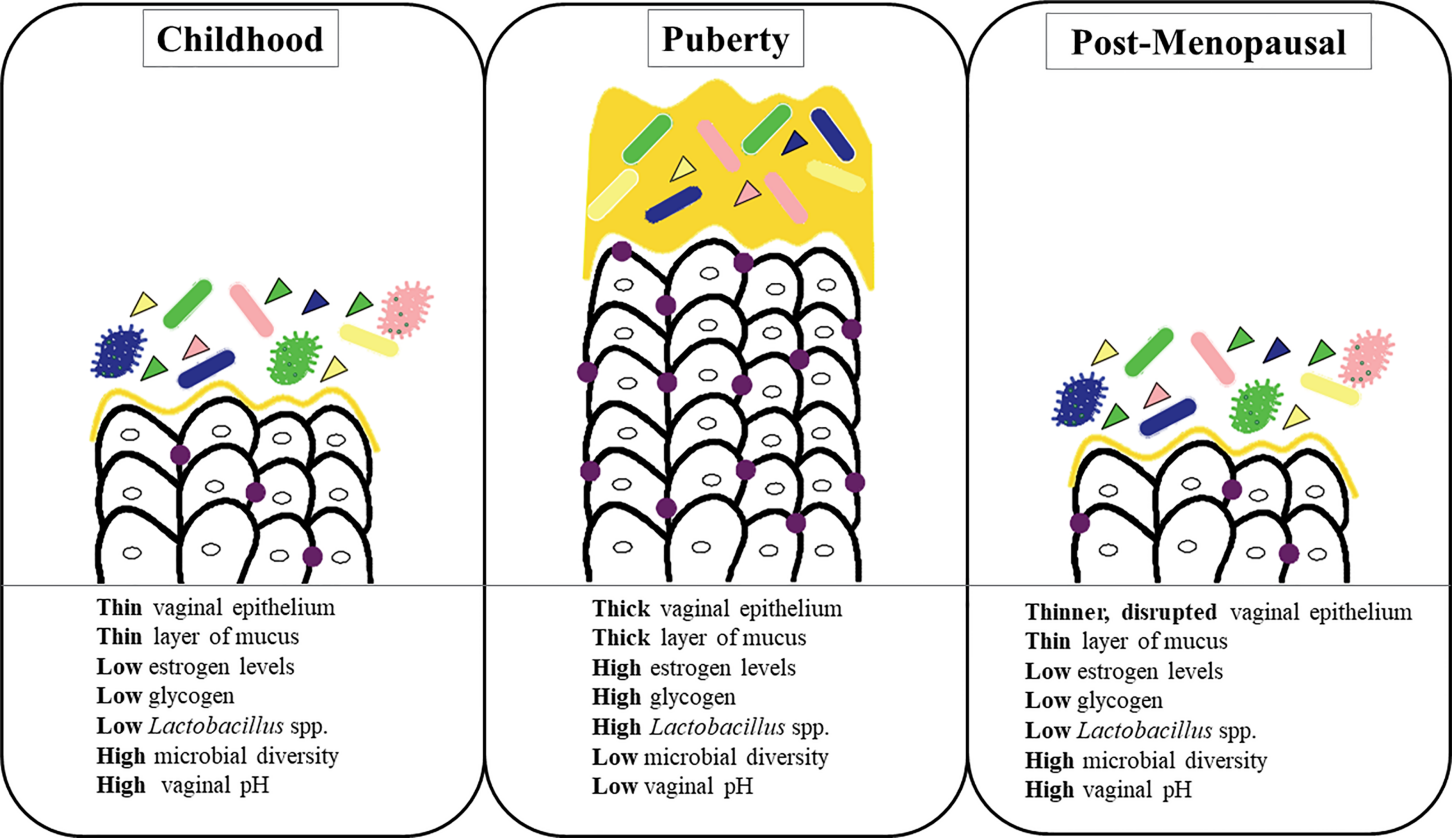
Radical change in the vaginal microbiome over a woman’s lifespan.

The composition of the vaginal microbiota evolves throughout a woman’s lifespan. Various physical and hormonal changes occur in the vagina biotope during these different stages of a woman’s life (Muhleisen and Herbst-Kralovetz, 2016; Nuriel-Ohayon et al., 2016) (**Figure 2**).

### 2.2 Variability of Vaginal Flora According to Ethnicity

Vaginal bacterial communities of women of childbearing age may vary between women from different regions, but also between women of different ethnicities living in the same geographical area (Hickey et al., 2012). In 2011, a study by Ravel et al. characterized the vaginal microbiota of asymptomatic North American women with pyrosequencing, showing that the vaginal flora of Asian and white American women was dominated by *Lactobacilli*, unlike Hispanic and African-American women, of whom only 60% had a *Lactobacillus* -dominated vaginal flora (Ravel et al., 2011). In addition, Caucasian and Asian women tend to have high levels of *L. crispatus* and lower levels of *L. iners* compared to African women (Green et al., 2015). In another study using 16S rRNA gene sequencing, Fettweis et al. demonstrated that the vaginal microbiota of European-ancestry women was dominated by *Lactobacilli*, as opposed to African-American women, who presented a mixed vaginal community containing, among others, *Mycoplasma hominis, Aerococcus*, *L. iners* and numerous strict anaerobes, including gram-positive anaerobic cocci, BV-associated bacteria, *Sneathia, Prevotella amnii, Megasphaera, Atopobium*, and *Gardnerella vaginalis* (Fettweis et al., 2014). The vaginal pH also differs between racial groups. African-American and Hispanic women had a vaginal pH (4.7 and 5.0, respectively) higher than what is considered the norm (<4.5) (Hickey et al., 2012).

### 2.3 Role of the Vaginal Microbiota in Women’s Health

The vaginal flora presents one of the most important defense mechanisms for reproductive function and maintaining a healthy environment. The stability of this flora prevents the proliferation of commensal microorganisms and colonization by pathogens, thereby preventing infection (Deidda et al., 2016; Donders et al., 2017). Bacteria form a adhered monolayer on the vaginal mucosa and produce antimicrobial compounds that maintain this health equilibrium, such as hydrogen peroxide (antimicrobial product protecting against deleterious microorganisms) (Cherpes et al., 2008; Sgibnev and Kremleva, 2015), lactic acid (which maintains the normal vaginal pH between 3.5 to 4.5) (O’Hanlon et al., 2013; Tachedjian et al., 2017), bacteriocins (antibiotics that inhibit the growth of harmful microorganisms within the vagina) (Stoyancheva et al., 2014), and arginine deaminase enzyme (metabolizes arginine into citrulline and ammonia (NH3), depriving anaerobic pathogens of this amino acid necessary for their growth**)** (Rousseau et al., 2005; Makarova et al., 2006).

Notably, *L. crispatus* and *L. jensenii* may produce hydrogen peroxide, an oxidizing agent, toxic for catalase-negative bacteria and also capable *in vitro* of inhibiting HIV-1 and herpes simplex virus type 2 (Aldunate et al., 2013; Borges et al., 2014). The vaginal acids produced can, in the presence of viral RNA, stimulate the maturation of dendritic cells, activation of 17 subclasses of T helper lymphocytes, and the production of protective inflammatory cytokines and interferon-γ (Witkin, 2015).

In addition to the role of *Lactobacilli*, cervical mucus is mainly composed of mucin, which protects the vaginal mucosa and optimizes its barrier role against microbial colonization. Analyses of the composition of cervical mucus and vaginal secretions have demonstrated the presence of several proteins with antimicrobial activities that act independently of the presence of antibodies, such as lactoferrin, lysosyme, calprotectin [also known as MRP8/MRP14 (“myeloid related protein”)], cathelicidin LL-37 (Nasioudis et al., 2017).

## 3 Bacterial Vaginosis

### 3.1 Background

**Figure 3:**
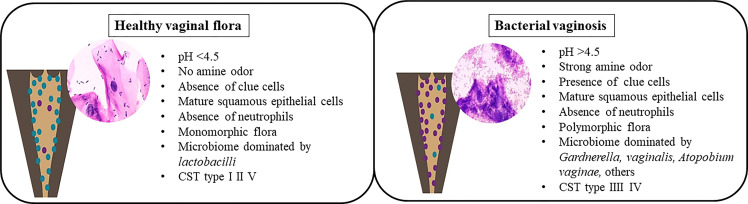
Healthy vaginal flora versus bacterial vaginosis.

Formerly known as non-specific vaginitis (Amsel et al., 1983), BV is characterized by a change in the vaginal flora composition, with a dramatic depletion of *Lactobacilli* due to a significant overgrowth of obligate or facultative anaerobes previously a minority in the vagina (Mårdh, 1993; Marrazzo and Hillier, 2013), such as *Gardnerella vaginalis, Atopobium vaginae, Ureaplasma urealyticum, Mycoplasma hominis, Prevotella, Peptoniphilus, Megasphaera, Mobiluncus*, and several fastidious and uncultured bacteria, including BV-associated bacteria (BVAB-1 to 3) (Romero et al., 2014; Margolis and Fredricks, 2015; Zozaya et al., 2016). The factor triggering this overgrowth of anaerobic bacteria is unknown. It is linked to an alkaline vaginal ecosystem due to an increase of vaginal pH following the loss of *Lactobacilli* protective effects (**Figure 3**).

The diversity of the vaginal flora in patients with BV was described in 1921 by Schröder (Kumar et al., 2011). In 1955, Gardner and Dukes claimed that the etiological agent of BV was *Haemophilus vaginalis* (Gardner and Dukes, 1955), a gram-negative rod later renamed *Gardnerella vaginalis* (Green et al., 2015; Mendling, 2016). The bacteria present in the microbiota of BV form a biofilm on the vaginal epithelium and secrete a cytotoxin capable of killing the epithelial cells (Huang et al., 2014). In addition, *G.vaginalis* produce proteolytic enzymes able to degrade proteins and decarboxylases that convert amino acids. Not being degraded, the amine compounds become malodorous (fishy odor: “Whiff test”) thank to an increase of the vaginal pH (Gonzalez Pedraza Aviles et al., 1999). Subsequently, cytotoxicity resulting from the combination of organic acids present in the vagina during BV and bacterial polyamines leads to the production of a vaginal discharge caused by the exfoliation of vaginal epithelial cells (Sobel, 2017). Furthermore, this bacterium, cover the vaginal epithelial cells, causing the formation of “clue-cells”, a specific characteristic of BV (Khan et al., 2007).

A few years later, *G. vaginalis* was found in 40% of healthy women, and its role was disputed (Machado et al., 2016). Thus, colonization of *G. vaginalis* does not always promote BV (Hickey and Forney, 2014), suggesting that this bacterium alone may be essential but not sufficient for BV development. In addition to *G. vaginalis*, some anaerobic bacteria are highly associated with BV, indicating that BV is a polymicrobial syndrome, which does not follow Koch’s postulates (Kumar et al., 2011).

Recent progress in research of BV pathogenesis have determined the existence of 13 different species within the genus *Gardnerella* (Vaneechoutte et al., 2019). Although these *Gardnerella* species may be closely linked genetically, only a few of them may be implicated in disease such as BV (Vaneechoutte et al., 2019). Healthy women may be colonized by non-pathogenic *Gardnerella* species, whereas virulent strains are involved in the development of BV. Advances in technology, particularly next-generation sequencing, have clarified much of this issue. Arguably, all papers dealing with *G. vaginalis* prior to that of Vaneechoutte et al. are not specifically about *G. vaginalis* but rather about *Gardnerella* spp (Vaneechoutte et al., 2019).

Based on a recent prospective study, an updated conceptual model on the pathogenesis of BV was outlined (Alves et al., 2014; Schellenberg et al., 2016; Castro et al., 2017; Muzny et al., 2019). The potential synergistic relationship between *G. vaginalis, P. bivia, A. vaginae* was studied (Muzny et al., 2018; Gilbert et al., 2019). After sexual exposure to virulent strains of *G. vaginalis*, these strains displace vaginal *Lactobacilli* and begin to form a BV biofilm on the vaginal epithelium (Machado and Cerca, 2015; Beebout et al., 2019). Subsequently, proteolysis by *G. vaginalis* occurs which promotes the growth of *P. bivia*. This bacterium produces an ammonia product which in turn promotes the growth of *G. vaginalis* and the biofilm develops (Gilbert et al., 2019; Castro et al., 2019). These 2 bacteria then produce sialidase that degrades the biofilm and *P. bivia* may thus degrade the mucin layer of the vaginal epithelium (Briselden et al., 1992; Gilbert et al., 2019). After the loss of the protective mucus layer, increased adhesion of other BV-associated bacteria, including *A. vaginae*, to the polymicrobial biofilm will occur (Hardy et al., 2016). The role of the other bacteria remains unknown (Muzny et al., 2019). Further research focusing on the complex interactions between bacteria during BV is needed.

### 3.2 Diagnosis

BV ranges from asymptomatic to an increase in vaginal discharge with or without a fishy odor (Gardner and Dukes, 1955; Majigo et al., 2021). The collection of a specimen for diagnosis can be performed using a speculum during the pelvic exam. When there is no reason for a pelvic exam as part of the clinical evaluation, a self-collected vaginal swab may also be provided (Menard et al., 2012; Camus et al., 2021).

#### 3.2.1 Amsel Criteria and Nugent’s Score

Two main categories of diagnostic strategies for BV exist: the “bedside” method introduced in 1983, mainly based on real-time clinical criteria –”Amsel’s criteria” (Amsel et al., 1983), and laboratory-based testing developed in 1991, relying on the evaluation of morphotypes on gram staining – “ Nugent’s score” (Nugent et al., 1991). Amsel’s criteria and Nugent’s score are the most common diagnostic methods used for BV. Furthermore, the World Health Organization (WHO) has considered the Nugent’s score as a gold standard for studies. However, the current recommended best clinical practice for diagnosing BV in women is gram staining microscopy according to the Hay-Ison criteria, as it is easier and faster to use (Sherrard et al., 2018). The Hay-Ison criteria were analogous to the Nugent’s scores. Hay’s grades I, II, and III were similar to Nugent’s scores 0-3, 4-6, and 7-10 (Ison and Hay, 2002).

Even if Nugent’s score is considered as a gold standard by the WHO, it has some pitfalls. In fact, intermediate flora is so far an uncharacterized category and is a challenge in the diagnosis of BV. In addition, the identification of morphotypes is subjective and technician-dependent, thus the diagnosis may be influenced by individual skills and experience (Menard et al., 2008; Antonucci et al., 2017).

Recently, a study made by Wang et al. provided a proof of concept for a deep learning-based model to quantify Gram staining and, consequently, automated Nugent score classification. Deep learning methods, particularly convolutional neural network (CNN) models, have demonstrated excellent performance in computer vision tasks. This model outperformed human healthcare professionals in terms of accuracy and stability for three diagnostic categories of Nugent scores. The deep learning model may offer translational applications in automating the diagnosis of BV with appropriate supporting hardware (Wang et al., 2020).

#### 3.2.2 Molecular Diagnostic Technique

The diagnosis of BV is problematic and challenging because of its intricate polymicrobial features and a wide range of clinical features (Money, 2005). In order to overcome these diagnostic problems, alternative diagnostic strategies have been attempted, such as molecular, enzymatic and chromatographic techniques.

A molecular technique used in the diagnosis of BV is specific quantitative real-time PCR (qPCR) test. It is a quantitative, reproducible and reliable molecular biology tool that measures the presence of bacteria presents in BV, such as *Atopobium vaginae, BVAB2, Gardnerella vaginalis, Leptotrichia/Sneathia* spp., *Megasphaera* spp., and *Mobiluncus* spp*….* (Breding et al., 2020). Many studies proposed objective molecular cut-off values from bacterial load to predict BV (Menard et al., 2008; Redelinghuys et al., 2017).

Several commercially molecular diagnostic assays have been reported for the diagnosis of BV in women including the NuSwab R multiplex quantitative PCR (Cartwright et al., 2012; Cartwright et al., 2018), SureSwab BV DNA real-time quantitative assay, BD Max vaginal panel (Gaydos et al., 2017) and BV multiplex assay (Hilbert et al., 2016). The quantification of these bacteria therefore makes it possible to establish a precise diagnosis of BV, with a sensitivity ranging from 90.5% to 96.7% and a specificity ranging from 85.8% to 95% compared to Amsel criteria and Nugent score (Coleman and Gaydos, 2018).

Even though these tests have a higher sensitivity and specificity than the currently available diagnostic tools, they are not point-of-care tests and are more expensive. However, Dessai et al. are the first to report on the performance of the BD AffirmTM VPIII test as a POCT in a prenatal population. But the test has been shown to be inadequate as a screening test for vaginal infections in pregnancy (Dessai et al., 2020).

Finally, POC tests for BV are not available or are simply too expensive to be used routinely. It is therefore mandatory that the development and evaluation of new diagnostic tests include a cost analysis.

#### 3.2.3 Other Emerging Strategies

As the presence of sialidase is currently considered a key indicator of BV in the clinical examination, an enzymatic approach has been developed: The OSOM R BVBlue R test as a POC diagnostic test for BV. It is based on the qualitative detection of a high level of sialidase produced by anaerobic pathogens in vaginal fluid samples. It has been shown to be reliable compared to conventional methods such as Amsel criteria and Nugent score (Myziuk et al., 2003; Shujatullah et al., 2010; Khatoon et al., 2013; Madhivanan et al., 2014)

In addition, a recent study by Liu et al., showed the feasibility of turn-on tetravalent sialic acid-coated tetraphenylethene luminogen (TPE4S) as a powerful diagnostic tool for high-throughput fluorescence-guided diagnosis of BV. This study uses light signal intensity to detect and measure the relative concentration of sialidase in a vaginal sample. All reagents are present in a reagent bead and sample buffer, essentially allowing for a one-step test. The test is highly sensitive and quantitative, with a sensitivity and specificity of 95.40% and 94.94%, respectively, compared to the Amsel method and 92.5% and 91.8% compared to the BVBlue diagnostic results. Notably, this method gives a more accurate classification and quantification of BV severity based on relative fluorescence intensity (I/I0). Thus this test can be a potential tool for diagnosis of BV, and risk assessment of patients with BV based on sialidase activity levels and monitoring of antibiotic therapy (Liu et al., 2018).

Another new approach based on immunodetection also targeting sialidase has been developed for the diagnosis of BV. The nanophotonic operating principle of this biodetection method allows a cheaper, faster and simpler analysis than the indirect enzyme-linked immunosorbent assay (ELISA). This nanotechnology has a high sensitivity and specificity (96,29%, respectively). This method offers an original approach to perform a very rapid diagnosis of BV (Rodríguez-Nava et al., 2021).

### 3.3 Epidemiology and Risk Factors

BV may appear at any reproductive age (between 15 to 44 years-old). Its prevalence rates vary considerably among the geographic regions of the world, within the same country, and even within the same population, depending on ethnic origin and socioeconomic status. Although its exact prevalence remains difficult to determine, BV occurs between 4-75%, depending on the population studied (Onderdonk et al., 2016; Bitew et al., 2017). Intermediate in the USA (29%), the prevalence of BV is estimated to be low in Europe, with a maximum (> 20%) in Poland and Norway (Kenyon et al., 2013). In Africa, the estimated prevalence tends to be high. However, BV prevalence is lowest in west Africa (6-8% in Burkina Faso, 14.2% in Nigeria) than southern and eastern Africa: 32.5% in Zimbabwe, 37% in Kenya, 38% in Botswana, and 68.3% in Mozambique (Kenyon et al., 2013; Afolabi et al., 2016; Bitew et al., 2017).

#### 3.3.1 Sexual Practices

Although the absence of a known causal agent makes it difficult to characterize BV as a sexually transmitted infection (STI) (Morris et al., 2001; Reid, 2018), it is strongly associated with sexual activities and has some characteristics of a sexually transmitted disease not by microorganism transfer, but by mechanical or chemical interaction such as contact with highly alkaline semen (Guédou et al., 2013; Muzny et al., 2013; Lewis et al., 2017). Overall, BV is diagnosed in post-pubertal women who have never been sexually active, but at a lower prevalence than those who are sexually active (Cherpes et al., 2008). The prevalence varies with the number of sex partners. It has been evaluated at 18.8% for non-sexually active women, 22.4% for women with one lifetime partner and 43.4% and 58% for women having 2-3 lifetime sex partners and those having ≥ 4-lifetime sex partners, respectively (Koumans et al., 2007).

In this dynamic, sex workers had a higher bacterial vaginal diversity but a much lower abundance of *Lactobacillus* species than women who are not engaged in sex work (Wessels et al., 2017). Compared with male partners of healthy women, BV-related bacteria can be found in the penile skin, urethra (Zozaya et al., 2016), spermatozoa, and prostatic fluid microbiota (Gallo et al., 2011; Hou et al., 2013) of male partners of women with BV. Furthermore, biofilm fragments have been found in their urine and sperm (Swidsinski et al., 2010a; Swidsinski et al., 2010b), suggesting that male partners are a reservoir, and also that heterosexual transmission may occur. Nevertheless, there is no corresponding illness in male partners (Verstraelen et al., 2010). Use of condoms by male partners also prevents acquisition and recurrence of BV (Verstraelen et al., 2010). Also, since the preputial area of some men hosts BV-associated microorganisms, male circumcision may reduce the risk of BV (Margolis and Fredricks, 2015).

Prevalence rates also depend on the nature of the couple and their sexual practices. In fact, BV prevalence varies between 10-30% in heterosexual women, and is more frequent, 25-50%, in women who have sex with women (WSW) (Forcey et al., 2015; Bilardi et al., 2016b). The reasons for this difference in prevalence are not clear, although sexual activities involving the transmission of vaginal fluid increase the risk of BV acquisition (Marrazzo and Hillier, 2013). Several studies have indicated that certain sexual behaviors, including non-coital sexual practices such as digital and penile penetration, anal and oral intercourse followed by vaginal penetration, enhance the risk of BV (Kenyon and Osbak, 2014). In WSW, a symptomatic female sexual partner, receptive oral sex, and the use and sharing of unwashed sex toys constitute risk factors for BV (Cherpes et al., 2008). These observations have led some to consider BV as not an infection, but rather a taxonomic change in the vaginal microbiota resulting from translocation of oral (Africa et al., 2014) or fecal (Fenollar and Raoult, 2016) microbiota during non-coital sexual practices.

#### 3.3.2 Other Bacterial Vaginosis Risk Factors

Additionally, genital hygiene can also promote disequilibrium in the vaginal microbiota. One study found that patients who did not wash their vaginal region were more susceptible to BV than those who often washed the vaginal region, a prevalence of 53.9% and 40.2%, respectively. Similarly, the prevalence of BV is higher in patients who do not change their underpants frequently compared to those who change it more frequently (57.6% versus 36.9%) (Bitew et al., 2017). In addition, other sexual sanitary habits, including vaginal douching and washing (Aslan and Bechelaghem, 2018), as well as cigarette smoking (Nelson et al., 2018), certain contraceptive methods like disposable intra-uterine devices (Achilles et al., 2018) and stress (Marrazzo and Hillier, 2013) may also enhance the risk of developing BV.

### 3.4 Bacterial Vaginosis Complications and Women’s Health

Women with BV are vulnerable: the presence of BV-related bacteria and/or sexually transmissible microorganisms in the BV microbiota can lead to opportunistic infections (Wiesenfeld et al., 2003; Brotman et al., 2014; Rumyantseva et al., 2019; Shipitsyna et al., 2020). During this imbalance, 10-30% of pregnant women with BV give birth prematurely, a preterm delivery often accompanied by perinatal mortality, up to 70% worldwide (Svare et al., 2006; Afolabi et al., 2016). During pregnancy, BV increases the risk of preterm labor, late miscarriage, intrauterine fetal death, preterm rupture of the membranes, amniotic fluid infections, chorioamnionitis, post-abortion and postpartum infections in these women (Wilson et al., 2011; Nelson et al., 2015a; Kairys and Garg, 2017; Brown et al., 2018).

In non-pregnant women, bacteria involved in BV can initially cause cervicitis, endometritis, salpingitis, and urinary tract infections (Georgijević et al., 2000). After damage of the cervix, bacteria can migrate from the lower to upper genital tract, reaching the uterus and fallopian tubes and causing illnesses such as pelvic inflammatory disease (Wilson et al., 2011; Sharma et al., 2014), post-hysterectomy infections (Marrazzo and Hillier, 2013), and even cervical cancer or tubal infertility (van van Oostrum et al., 2013; Babu et al., 2017). Likewise, BV is associated with significantly increased rates of acquiring herpes simplex virus (Nardis et al., 2013), human immunodeficiency virus (McClelland et al., 2018), papillomavirus (Kero et al., 2017) and transmission of the pathogens causing syphilis, chancroid, gonorrhea, trichomoniasis, and chlamydia (Brotman, 2011; Bitew et al., 2017).

### 3.5 Treatment and Management of Bacterial Vaginosis

Considering that clinical cure corresponds to the disappearance of all symptoms, the treatment of BV is currently focused on stopping the proliferation of BV-associated microorganisms and restoring the normal vaginal flora (Marrazzo and Hillier, 2013). Typically, clinical therapies include the use of antibiotics with broad activity against anaerobic microbes and protozoa: clindamycin and the nitroimidazoles (metronidazole and tinidazole) and/or use of probiotics (Kumar et al., 2011; Bacterial Vaginosis, 2015; Bradshaw and Sobel, 2016).

#### 3.5.1 Antibiotic Therapies

The first line of therapy recommended by the World Health Organization (WHO) is 500 mg oral metronidazole twice daily for one week (Bacterial Vaginosis, 2015; World Health Organization, 2021). However, treatment with metronidazole may cause side effects such as gastrointestinal pain, nausea, and vomiting (Machado et al., 2016). Other proposed therapeutic regimens include 300 mg oral clindamycin twice daily for one week, 100 mg of intravaginal clindamycin ovule daily for 5 days and an application of 0.75% intravaginal metronidazole gel for 5 days or 2% of intravaginal clindamycin cream at bedtime for one week (Donders et al., 2014; World Health Organization, 2021). However, it should be noted that local application of clindamycin may damage latex-based products such as condoms and may also trigger pseudomembranous colitis (Machado et al., 2016).

In addition, the use of tinidazole, a drug similar to metronidazole, has been approved and proposed as an alternative therapy in an oral regimen (either 2 g/day for 2 days or 1g/day for 5 days) if metronidazole and clindamycin are not tolerated (Bacterial Vaginosis, 2015; Dickey et al., 2009).

Some researchers have evaluated the efficacy of other antimicrobial agents, such as azithromycin, secnidazole or ornidazole and rifaximin (Schwebke and Desmond, 2007; Thulkar et al., 2012; Laghi et al., 2014). Secnidazole has shown activity similar to that of the recommended nitroimidazoles and also spares *Lactobacilli*, a beneficial characteristic in BV treatment. Even for Rifaximin, it acts on BV by restoring *Lactobacilli* and increasing lactic acid in patients (Bagnall and Rizzolo, 2017).

Taken locally or orally, these antimicrobial agents have almost similar efficacy, with cure rates around 58% to 92% after 1 month of treatment (Donders et al., 2014). Nevertheless, these results are temporary, leading to recurrence or re-infection at rates above 50% within 6-12 months of treatment (Bilardi et al., 2016a; Bradshaw and Sobel, 2016). The reasons for this high relapse rate remain unclear. However, it appears that, with the formation of bacterial biofilms, these recommended therapies only temporarily eradicated BV-associated microorganisms, or these bacteria are reintroduced in the vagina by their sex partners (Marrazzo et al., 2012; Bradshaw and Brotman, 2015; Margolis and Fredricks, 2015). Further, the presence of some BV-associated bacteria such as *Peptoniphilus lacrimalis, Megasphaera* type 2 and BVAB-1 to 3 at the beginning of treatment is strongly related to BV recurrence, thus causing antibiotic failure (Marrazzo et al., 2008). To this end, two recent studies have examined the acceptability, tolerability, and especially the efficacy of concomitant partner treatment to improve the cure of BV (Schwebke et al., 2021; Plummer et al., 2021). The first study by Schwebke et al, showed no significant reduction in BV recurrence in female partners after treatment of the male partner with multidose metronidazole, although women whose partners adhered to multidose metronidazole were less likely to fail treatment (Schwebke et al., 2021). The second study showed that simultaneous partner treatment had a significant change in the overall composition of genital microbiota in both partners immediately after treatment (Plummer et al., 2021).

#### 3.5.2 Non-Antibiotic Therapies

As antibiotic treatments can have a negative impact on the stability of the vaginal flora, *Lactobacillus* probiotics, an alternative and complementary therapy to antibiotic treatment, has been developed to help restore and maintain the healthy vaginal flora (Bradshaw et al., 2012). Probiotics consist of living microorganisms that confer a health benefit on the host when they are administered in an appropriate quantity (Borges et al., 2014). Nine studies from 1989 to 2014 tested the effectiveness of vaginal or oral *Lactobacillus* probiotics (Fredricsson et al., 1989; Hallén et al., 1992; Wewalka et al., 2002; Mastromarino et al., 2009; Hummelen et al., 2010; Hemalatha et al., 2012; Ling et al., 2013; Vujic et al., 2013; Vicariotto et al., 2014). The results showed that both oral and vaginal *Lactobacillus* treatments were effective in curing acute BV. On balance, the application of *Lactobacillus* in the form of vaginal capsules (containing≥10^8^ CFU of *Lactobacillus* strains per dose) or a fermented milk product (containing≥5×10^9^ CFU of *Lactobacillus* strains per dose) may be an equally effective alternative to standard antibiotic capsules. Only the strains *L. reuteri* RC-14 and *L. rhamnonus* GR-1 have positive clinical effects (Kumar et al., 2011; Ouarabi et al., 2017). Administrated orally (twice daily) or vaginally (once weekly), probiotics may restore the normal *Lactobacillus*-dominated microbiota and reduce BV recurrence (Daliri and Lee, 2015). Nevertheless, probiotics have had minimal success in African women (Margolis and Fredricks, 2015).

In the same context, a new study on the applicability of three strains of *Lactobacillus* spp. (*Lactobacillus delbrueckii* DM8909, *Lactiplantibacillus plantarum* ATCC14917 and *Lactiplantibacillus plantarum* ZX27) according to their *in vitro* probiotic capacities. These three *Lactobacillus* spp. strains have shown efficacy in the treatment of BV by limiting the growth, adhesion, biofilm formation and virulence properties of *G. vaginalis* (Qian et al., 2021).

In addition, sucrose-containing products may promote recolonization, since sucrose is metabolized by *Lactobacilli*. A triple-blind randomized clinical trial compared the efficacy of a sucrose vaginal gel versus a metronidazole vaginal gel for 5 days in 70 women with diagnosed BV (Khazaeian et al., 2018). The sucrose vaginal gel was as effective as metronidazole treatment, according to this individual RCT.

Another option is to combine *Lactobacilli* with estriol. Two studies, a PC-RCT and a head-to-head RCT, were conducted on the efficacy of vaginal capsules containing *L. acidophilus* and 0.03 mg estriol (Gynofor^®^) in BV (Parent et al., 1996; Donders et al., 2010). Compared to placebo, the cure rate of BV was significantly higher in the treated group, but according to the comparative study, the combination of estriol and *Lactobacilli* is equivalent, but not better, results than antibiotic treatment.

In terms of method of application, vaginal suppositories deposit *Lactobacillus* strains directly on the vaginal mucosa, whereas oral probiotics survive gastrointestinal transit and increase the number of strains in the colon and feces. In turn, this may promote recolonization of the vagina due to the proximity of the rectum and vagina (Tidbury et al., 2020).

#### 3.5.3 New Emerging Therapies

The management of BV urgently requires implementation of new therapeutic strategies. To disrupt BV-associated biofilms, studies are underway to investigate the role of novel agents such as DNases, retrocyclines, antiseptics, and plant-derived compounds in the treatment of BV (Machado et al., 2016). Dequalinium chloride, an antiseptic, has reported similar efficacy to clindamycin intravaginal cream (Weissenbacher et al., 2012). Thymol, a molecule found in thyme essential oil, has shown an inhibitory effect on biofilms *in vitro* (Braga et al., 2010). The application of acidifying agents, such as vitamin C or buffering agents (polycarbophil or boric acid), in combination with a nitroimidazole antibiotic, has been demonstrated to reduce the recurrence of BV, potentially by destructuring the vaginal biofilm (Machado et al., 2016).

In this regard, other promising therapeutic agents for the treatment of BV are under investigation. Including DNase agents that can disrupt vaginal biofilms by targeting extracellular DNA essential for their structural integrity (Hymes et al., 2013), retrocyclin 101, a synthetic cyclic antimicrobial peptide that inhibits the growth and development of *G. vaginalis in vitro* (Hooven et al., 2012), and the amphoteric tenside pessary (WO3191), which disrupts biofilms after metronidazole treatment and promotes the growth of *Lactobacillus* species (Gottschick et al., 2017; Algburi et al., 2017).

Furthermore, given the success demonstrated by fecal microbiota transplant (FMT) in the management of various intestinal disorders and diseases such as recurrent *Clostridium difficile* infection, pseudomembranous colitis, inflammatory bowel disease (IBD) and irritable bowel syndrome (IBS), the use of vaginal microbiota transplantation (VMT) in BV treatment is a new therapeutic approach that modulates the vaginal microbiota in order to eradicate this scourge and reduce adverse gynecological outcomes (DeLong et al., 2019).

Given the high rates of recurrence and relapse, research is needed to identify and evaluate these novel biofilm-disrupting treatment strategies.

## 4 Conclusions and Perspectives

The taxonomic composition and bacterial proportion of the vaginal microbiota are under the influence of intrinsic and external factors over the female lifespan. In the last decades, understanding of the bacterial diversity of this ecosystem has been increased by molecular methods. Dominated by *Lactobacilli* that protect against infection, the vaginal flora of healthy women is less complex than that of patients with BV, which presents a diverse microbiota containing numerous obligate anaerobic and uncultivable species. This polymicrobial condition is associated with relatively uncomplicated clinical symptoms that do not occur in all affected women, thus complicating the determination of its etiology. Treatment is usually unsuccessful, with a high rate of relapse. Future studies that thoroughly examine the vaginal bacterial community are needed to cultivate the bacteria associated with BV and its treatment failure, in order to study antibiotic resistance and to establish more effective alternative therapeutic strategies that reduce BV symptoms as well as its associated complications. Overall, unlocking the enigma of the pathogenesis of BV is key for the prevention and management of this public health problem.

## Author Contributions

LAC, FF, and KD have equally contributed to the preparation of this manuscript. All authors contributed to the article and approved the submitted version.

## Funding

This study was funded by the Institut Hospitalo-Universitaire (IHU) Méditerranée Infection, the National Research Agency under the “Investissements d’avenir” programme, reference ANR-10-IAHU-03, the Region Provence Alpes Côte d’Azur and European FEDER PRIMI funding.

## Conflict of Interest

The authors declare that the research was conducted in the absence of any commercial or financial relationships that could be construed as a potential conflict of interest.

## Publisher’s Note

All claims expressed in this article are solely those of the authors and do not necessarily represent those of their affiliated organizations, or those of the publisher, the editors and the reviewers. Any product that may be evaluated in this article, or claim that may be made by its manufacturer, is not guaranteed or endorsed by the publisher.
